# Author Correction: Apoptotic stress-induced FGF signalling promotes non-cell autonomous resistance to cell death

**DOI:** 10.1038/s41467-024-46206-x

**Published:** 2024-02-28

**Authors:** Florian J. Bock, Egor Sedov, Elle Koren, Anna L. Koessinger, Catherine Cloix, Désirée Zerbst, Dimitris Athineos, Jayanthi Anand, Kirsteen J. Campbell, Karen Blyth, Yaron Fuchs, Stephen W. G. Tait

**Affiliations:** 1 Cancer Research UK Beatson Institute, Garscube Estate, Switchback Road, Glasgow, G61 1BD UK; 2 Institute of Cancer Sciences, University of Glasgow, Garscube Estate, Switchback Road, Glasgow, G61 1QH UK; 3 Department of Radiotherapy (MAASTRO), GROW-School for Oncology and Developmental Biology, Maastricht University, 6229 ER Maastricht, The Netherlands; 4 Laboratory of Stem Cell Biology and Regenerative Medicine, Department of Biology, Technion Israel Institute of Technology, Haifa, 3200003 Israel

**Keywords:** Apoptosis, Cell signalling

Correction to: *Nature Communications* 10.1038/s41467-021-26613-0, published online 12 November 2021

The original version of this article contained an error in Figure 3e in which there are additional 3 western blot lanes that should have been omitted. This has been corrected in both the PDF and the HTML versions of the article.

