# Supplementary material for: Apoptotic stress-induced FGF signalling promotes non-cell autonomous resistance to cell death
Source: Nat Commun. 2021 Nov 12;12:6572. doi: 10.1038/s41467-021-26613-0 (PMC8590049; doi:10.1038/s41467-021-26613-0)
Supplement: Supplementary file 1 — Supplementary Information [file 41467_2021_26613_MOESM1_ESM.pdf]

Supplementary Figure 1

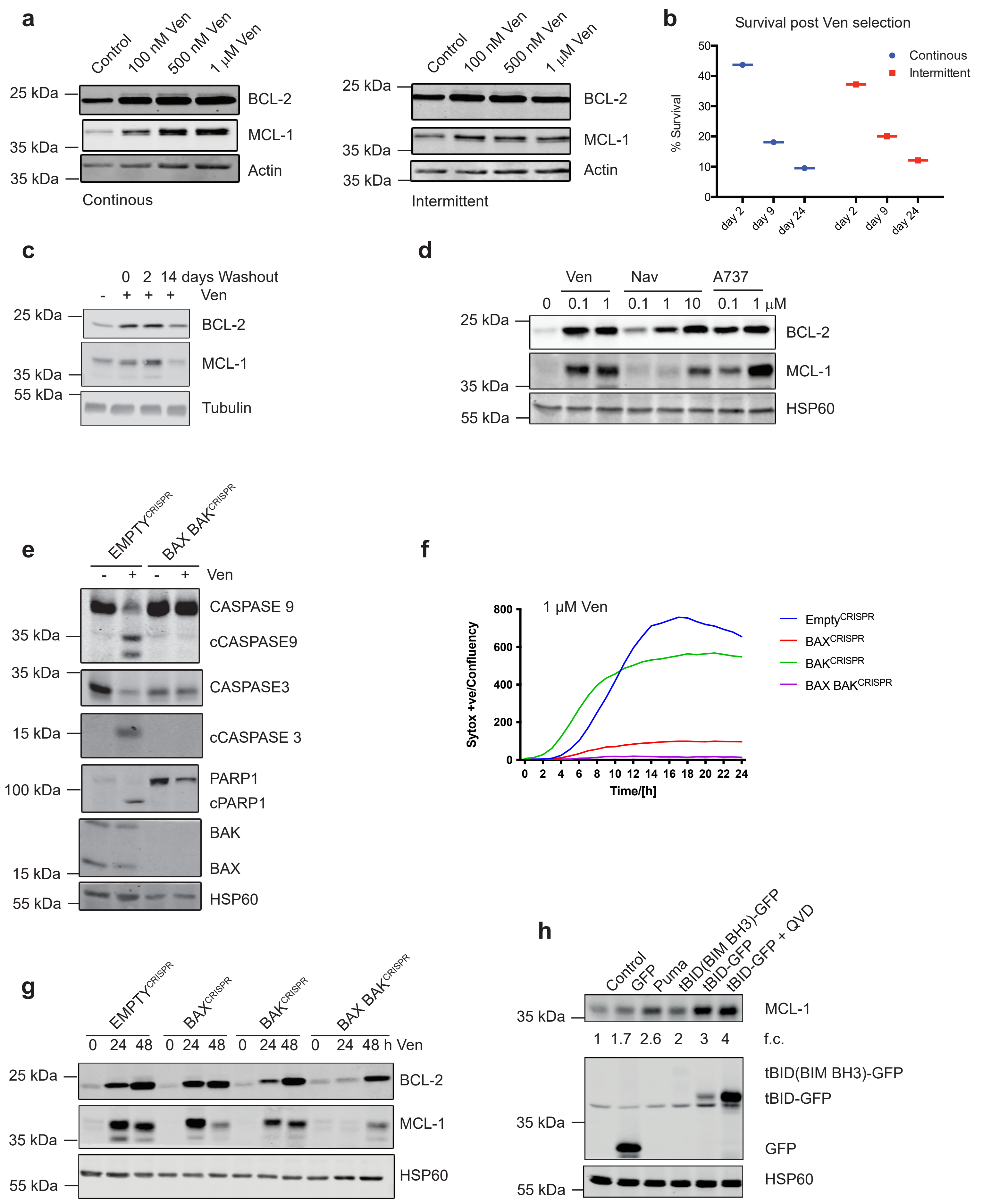

Supplementary Figure 1: BH3-mimetics and BH3-only proteins upregulate BCL-2 and MCL-1 causing apoptotic resistance

(a) HeLa tBID2A cells were continuously (left) or intermittently for 8 h daily (right) cultured in different doses of venetoclax for 14 days, after which cells were harvested and protein expression determined by western blot (representative blot of two independent repeats).

(b) HeLa tBID2A cells made resistant as described in (a) were cultured in regular medium for the indicated times and apoptosis was determined by flow cytometry with Annexin V/propidium iodide staining.

(c) HeLa tBID2A cells were treated for 48 h with 500 nM venetoclax followed by culture in regular medium as indicated. Cells were harvested, and protein expression determined by western blot (representative blot of three independent repeats).

(d) HeLa tBID2A cells were treated for 48 h with the indicated BH3-mimetics, harvested and analysed for protein expression by western blot (representative blot of two independent repeats).

(e) Control and BAX BAK^{CRISPR} HeLa tBID2A cells were treated with 500 nM venetoclax for 3 h, harvested and analysed for protein expression by western blot (representative blot of two independent repeats).

(f) Control, BAX^{CRISPR}, BAK^{CRISPR} and BAX BAK^{CRISPR} HeLa tBID2A cells were treated with 1 μM venetoclax and cell viability was monitored by Sytox green staining and Incucyte live-cell imaging.

(g) Control, BAX^{CRISPR}, BAK^{CRISPR} and BAX BAK^{CRISPR} HeLa tBID2A cells were treated with 500 nM venetoclax, harvested at the indicated time points and protein expression analysed by western blot (representative blot of three independent repeats).

(h) HeLa cells were transfected with the indicated constructs for 48 h and protein expression was analysed by western blot. Fold change normalised to loading control is stated below (representative blot of two independent repeats).

Supplementary Figure 2

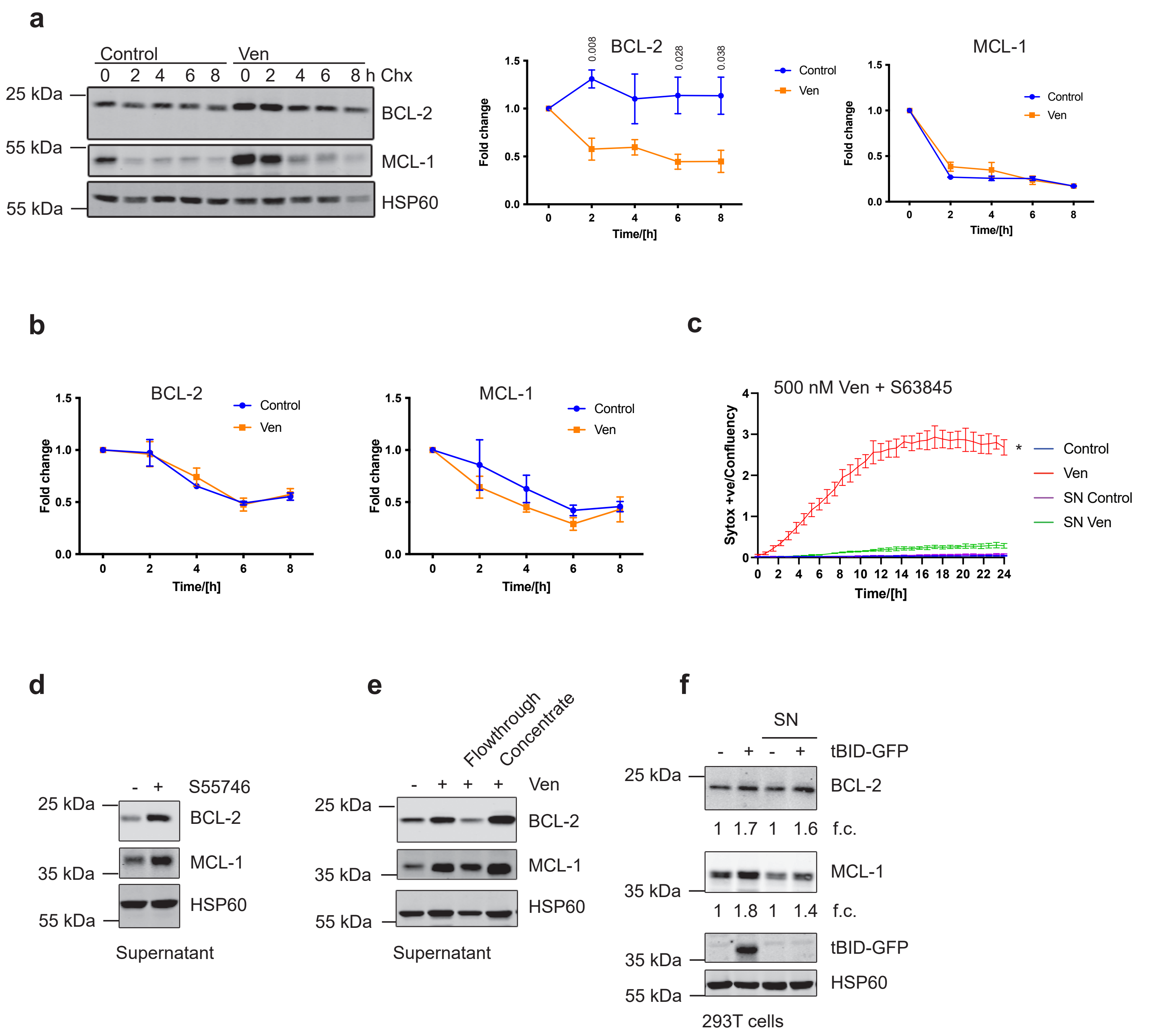

Supplementary Figure 2: BH3-mimetics and BH3-only proteins can upregulate anti-apoptotic BCL-2 proteins in a non-cell autonomous manner

(a) HeLa tBID2A cells were treated with 500 nM venetoclax for 24 h, followed by treatment with 1 μg/ml cycloheximide (Chx). Cells were harvested at the indicated times and protein expression analysed by western blot. Quantification normalised to HSP60 relative to t = 0 shown on the right. n = 3 independent experiments; mean values ± s.e.m.; unpaired, two-sided t-test.

(b) HeLa tBID2A cells were treated with 500 nM venetoclax for 24 h, followed by treatment with 1 μM actinomycin D. Cells were harvested for the indicated times and RNA expression analysed by RT-qPCR. n = 3 independent experiments; mean values ± s.e.m.

(c) HeLa tBID2A cells were treated as indicated and viability monitored by Sytox green uptake and Incucyte live cell imaging. n = 3 independent experiments; mean values ± s.e.m.; * $p < 0.0001$ at 24 h; Tukey corrected one-way ANOVA.

(d) Supernatant from control or S55747 (100 nM) treated HeLa tBID2A cells was added to recipient cells and protein expression was analysed by western blot after 48 h (representative blot of three independent repeats).

(e) Supernatant from control or venetoclax treated HeLa tBID2A cells was harvested and filtered with a 3 kDa cut-off centrifugal spin column. The concentrate was subsequently adjusted to its original volume and flowthrough and concentrate was added onto recipient cells. After 48 h, cells were harvested and protein expression analysed by western blot (representative blot of two independent repeats).

(f) 293T cells were transfected with 1 μg tBID-GFP for 24 h or treated with supernatant (SN) from tBID-GFP transfected 293T cells. After 24 h, the cells were harvested and protein expression was analysed by western blot. Fold change normalised to loading control is stated below (representative blot of three independent repeats).

Supplementary Figure 3

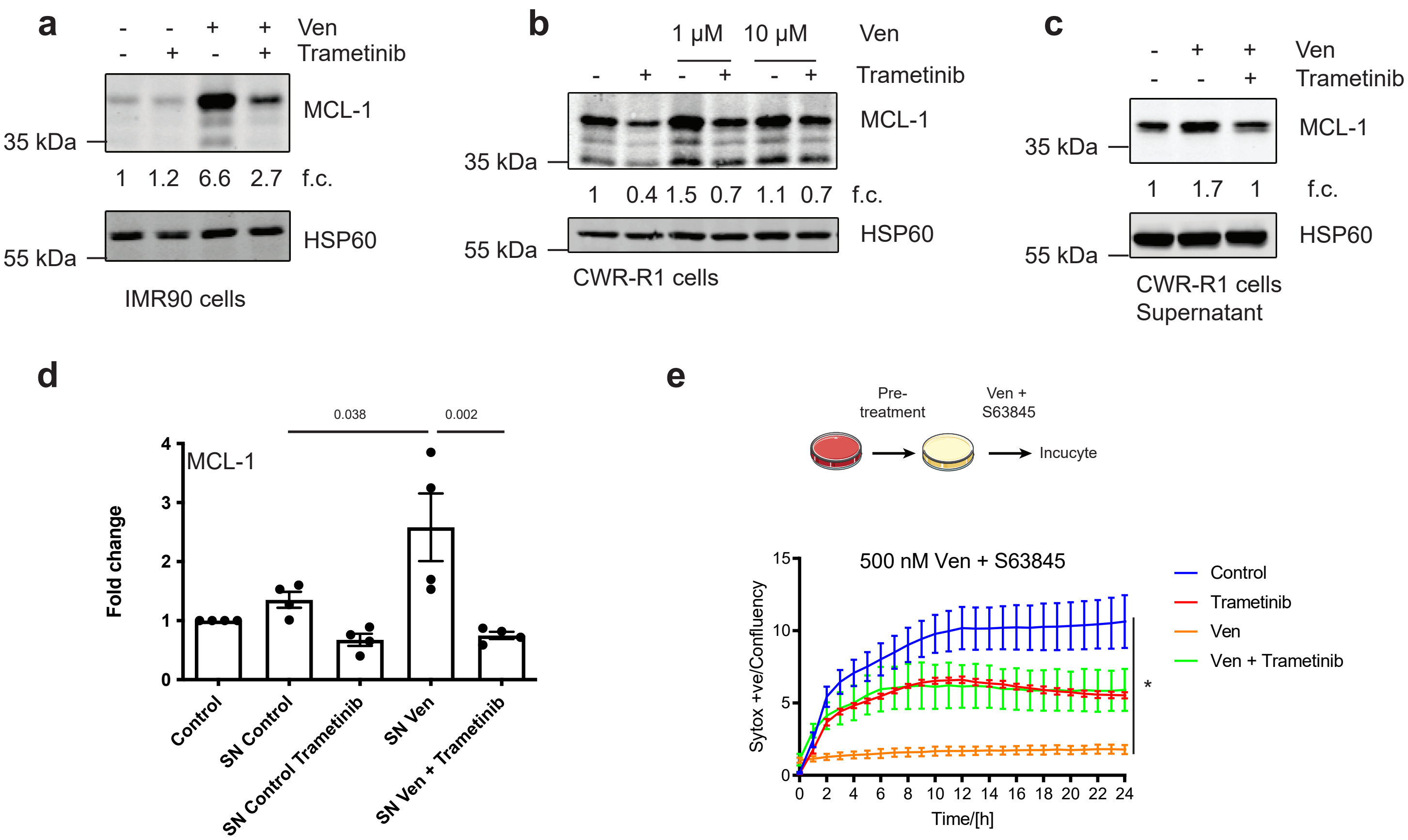

Supplementary Figure 3: Non-cell autonomous upregulation of anti-apoptotic BCL-2 proteins requires MEK-ERK signalling

(a) IMR90 cells were treated with 10 μM venetoclax in combination with trametinib as indicated. After 48 h, cells were harvested and protein expression analysed by western blot. Fold change normalised to loading control is stated below (representative blot of two independent repeats).

(b) CWR-R1 cells were treated with venetoclax in combination with trametinib as indicated. After 48 h, cells were harvested and protein expression analysed by western blot. Fold change normalised to loading control is stated below (representative blot of two independent repeats).

(c) Supernatant from control or venetoclax treated CWR-R1 cells was supplemented with 500 nM trametinib as indicated before addition onto recipient cells and protein expression was analysed by western blot after 48 h. Fold change normalised to loading control is stated below (representative blot of two independent repeats).

(d) Supernatant from control or venetoclax treated HeLa tBID2A cells was supplemented with 500 nM trametinib as indicated before addition onto recipient cells and RNA expression was analysed by RT-qPCR after 48 h. n = 4 independent experiments; mean values ± s.e.m.; Tukey corrected one-way ANOVA.

(e) HeLa tBID2A cells were treated with or without venetoclax in combination with 500 nM trametinib as indicated for 48 h followed by treatment with venetoclax and S63845. Cell viability was then monitored by Sytox Green staining and Incucyte imaging. n = 3 independent experiments; mean values ± s.e.m.; *: $p < 0.0001$ compared to venetoclax treatment at 24 h; Tukey corrected one-way ANOVA.

Supplementary Figure 4

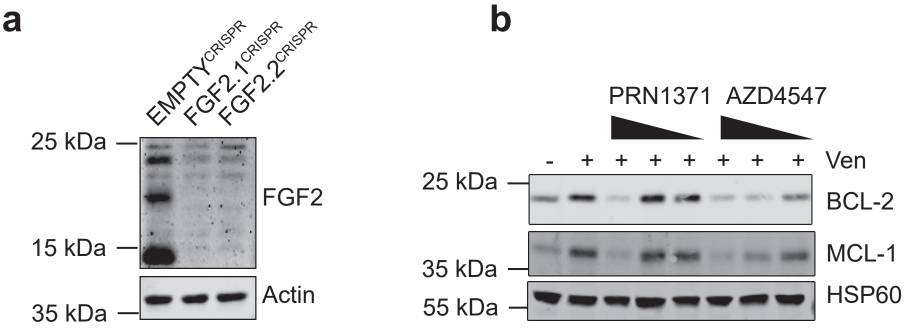

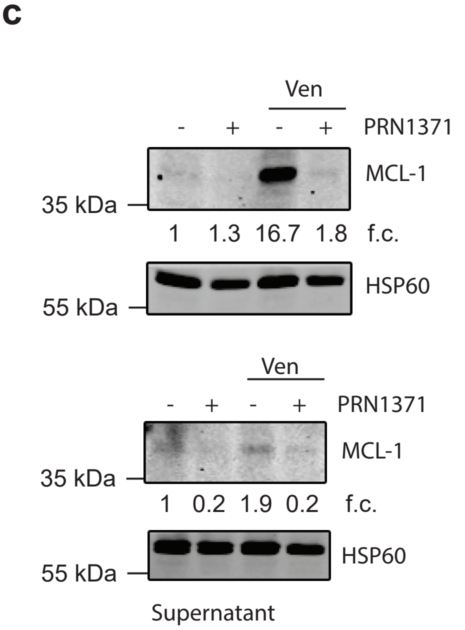

d

Gene	RPKM		
	Repeat 1	Repeat 2	Repeat 3
FGFR1	1026	1290	1045
FGFR2	16	13	14
FGFR3	226	301	295
FGFR4	420	365	396

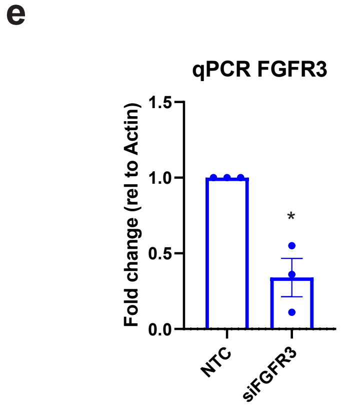

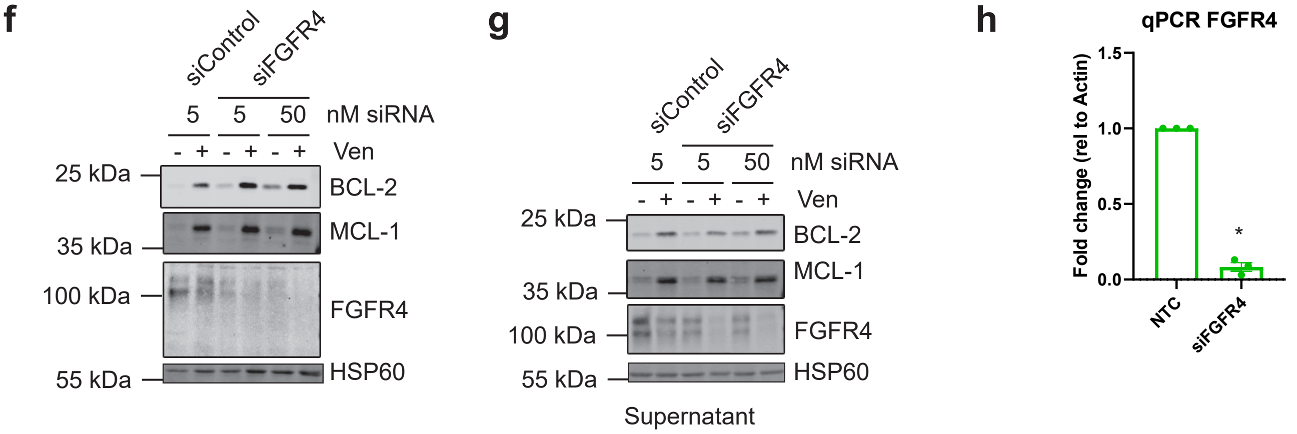

Supplementary Figure 4: FGF signalling mediates non-cell autonomous upregulation of BCL-2 proteins

(a) FGF2 deficient HeLa tBID2A cells were created via CRISPR-Cas9 genetic engineering with two independent guide RNAs and validated by western blot (representative blot of three independent repeats).

(b) HeLa tBID2A cells were treated with venetoclax in combination with decreasing doses of FGFR inhibitors as indicated (AZD: 5 μM, 2.5 μM, 1.25 μM; PRN1371: 10 μM, 5 μM, 2.5 μM), cells were harvested after 48 h and protein expression was analysed by western blot (representative blot of three independent repeats).

(c) MRC-5 cells were treated with venetoclax in combination with 10 μM PRN1371 (left) or with supernatant from MRC-5 cells treated with venetoclax and supplemented with 10 μM PRN1371 (right) as indicated for 48 h, after which cells were harvested and protein expression was analysed by western blot. Fold change normalised to loading control is stated below (representative blot of two independent repeats).

(d) Table showing reads per kilo base per million mapped reads (RPKM) values from FGF receptors in HeLa tBID2A cells.

(e) qPCR for FGFR3 from HeLa tBID2A cells transfected with control siRNA (NTC) or siRNA targeting FGFR3 for 48 h. n = 3 independent experiments; mean values ± s.e.m.; *: $p < 0.0001$; unpaired, two-sided t-test.

(f) HeLa tBID2A cells were transfected with the indicated amount of siRNA targeting FGFR4 for 24 h before treatment with 500 nM venetoclax for 48 h and analysis by western blot (representative blot of three independent repeats).

(g) HeLa tBID2A cells were transfected with the indicated amount of siRNA targeting FGFR4 for 24 h before addition of supernatant from control cells treated with 500 nM venetoclax for 48 h and analysis by western blot (representative blot of three independent repeats).

(h) qPCR for FGFR4 from HeLa tBID2A cells transfected with control siRNA (NTC) or siRNA targeting FGFR4 for 48 h. $n = 3$ independent experiments; mean values ± s.e.m.; *: $p < 0.0001$; unpaired, two-sided t-test.

Supplementary Figure 5

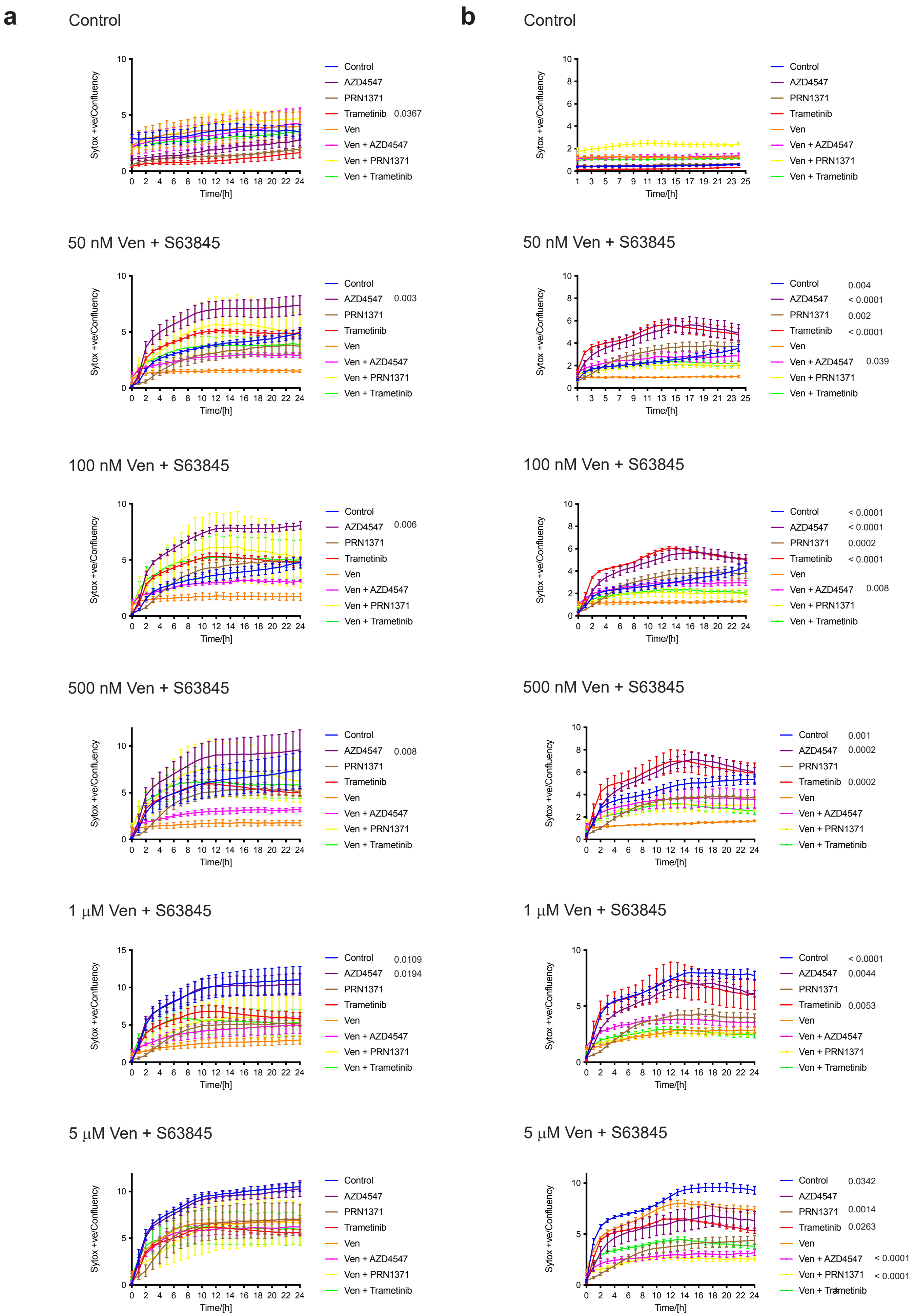

Supplementary Figure 5: FGF signalling is essential for non-cell autonomous apoptotic resistance

(a) HeLa tBID2A cells were treated with 500 nM venetoclax in combination with RTK pathway inhibitors (AZD4547 (5 μM), PRN1371 (10 μM) or trametinib (500 nM)) as indicated for 48 h. Then the cells were treated with venetoclax + S63845 and cell survival was monitored by Incucyte. n = 3 independent experiments; mean values ± s.e.m.; p values next to legend compared to venetoclax treatment at 24 h; Dunnett's corrected one-way ANOVA.

(b) HeLa tBID2A cells were incubated with 500 nM venetoclax treated supernatant supplemented with RTK pathway inhibitors (AZD4547 (5 μM), PRN1371 (10 μM) or trametinib (500 nM)) as indicated before addition onto recipient cells for 48h. Then the cells were treated with venetoclax + S63845 and cell survival was monitored by Incucyte. n = 3 independent experiments; mean values ± s.e.m.; p values next to legend compared to venetoclax treatment at 24 h; Dunnett's corrected one-way ANOVA.

Supplementary Figure 6

a Chromophobe renal cell carcinoma

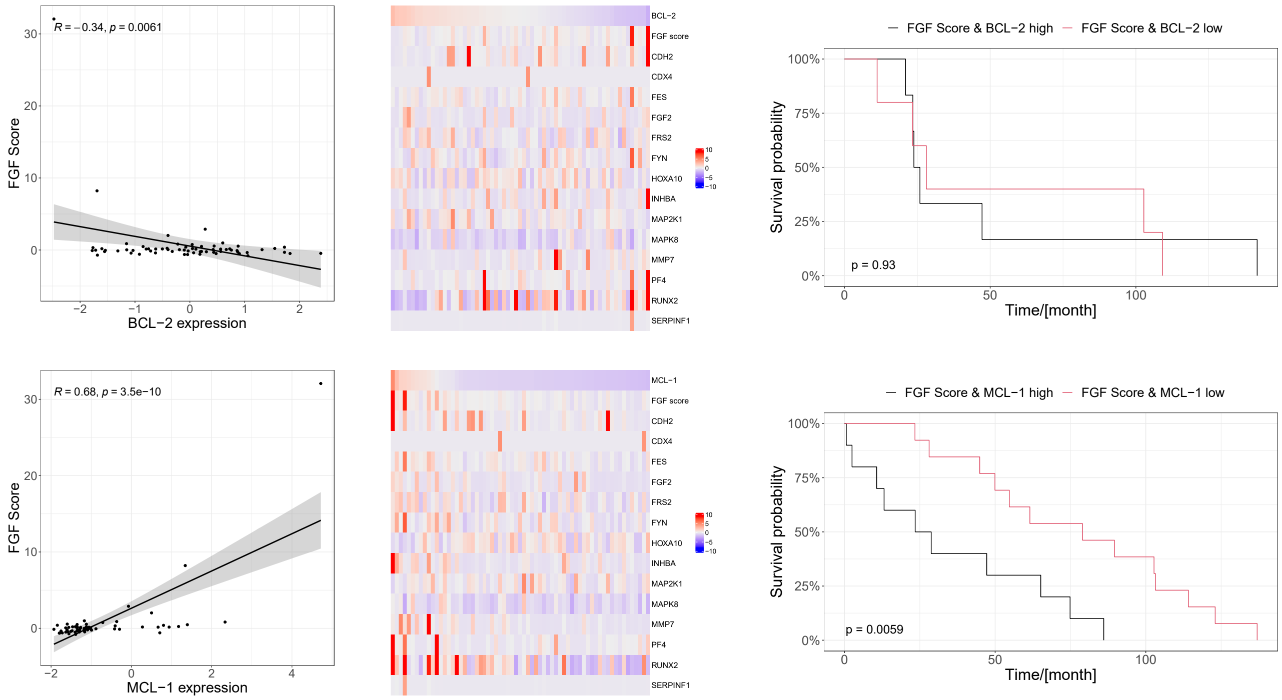

b Uterine corpus endometrial carcinoma

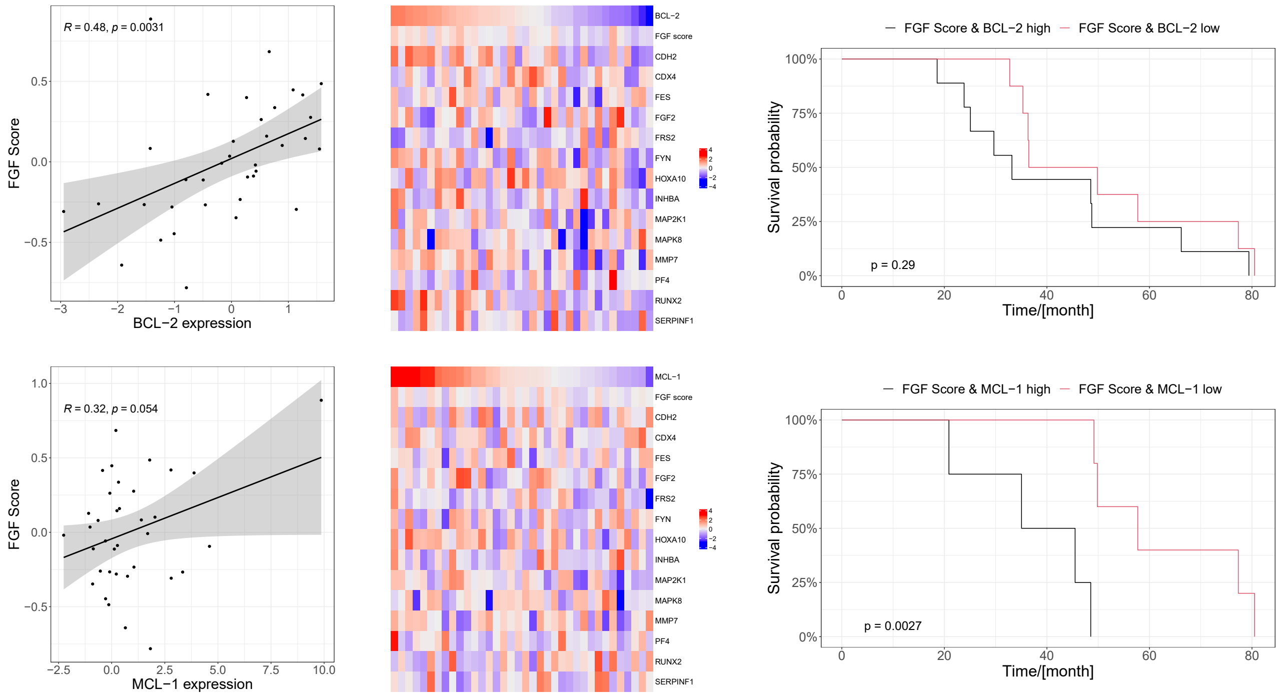

Supplementary Figure 6: Correlation between FGF signalling and BCL2 family member expression predict patient survival in some cancer types

(a) Pearson correlation between FGF Score and BCL-2 (upper left) or MCL-1 (lower left) in the TCGA chromophobe renal cell carcinoma dataset (± s.e.m). FGF score, FGF receptor target gene and BCL-2 (upper middle) or MCL-1 (lower middle) expression in the TCGA chromophobe renal cell carcinoma dataset. Survival of TCGA chromophobe renal cell carcinoma patients stratified by FGF score and BCL-2 (upper right) or MCL-1 (lower right) expression (p value was calculated with a log-rank test).

(b) Pearson correlation between FGF Score and BCL-2 (upper left) or MCL-1 (lower left) in the TCGA uterine corpus endometrial carcinoma dataset (± s.e.m). FGF score, FGF receptor target gene and BCL-2 (upper middle) or MCL-1 (lower middle) expression in the TCGA uterine corpus endometrial carcinoma dataset. Survival of TCGA uterine corpus endometrial carcinoma patients stratified by FGF score and BCL-2 (upper right) or MCL-1 (lower right) expression (p value was calculated with a log-rank test).

Supplementary Figure 7

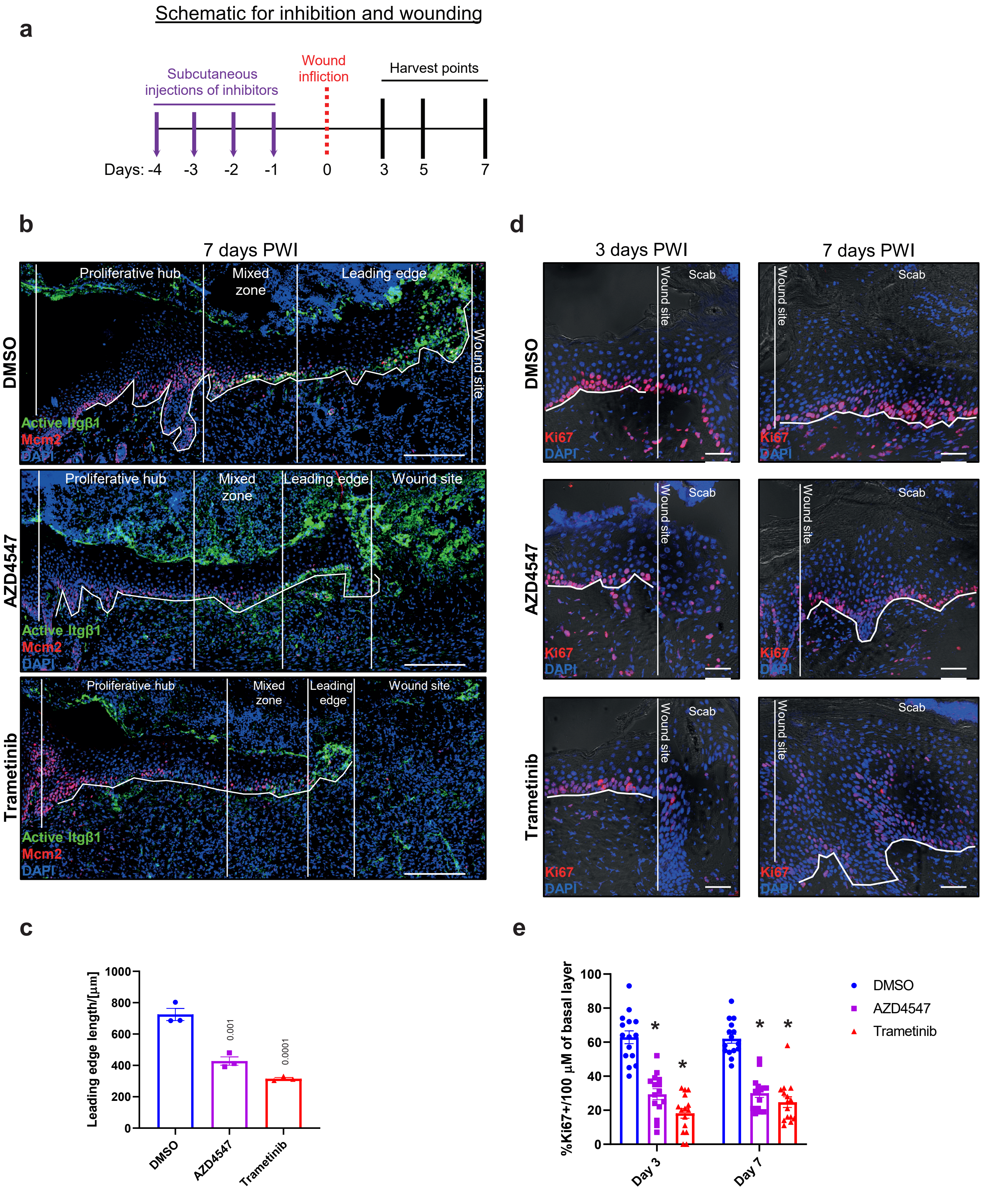

Supplementary Figure 7: FGFR and MEK inhibition decreases MCL-1 expression and hinders wound repair

(a) Schematic depiction of inhibition and wound healing experiments in mice.

(b) Mcm2 and active Integrin beta-1 immunostaining in control or inhibitor treated mouse skins at the wound site at 7 days PWI. Representative confocal images of n = 3 mice, Scale bar: 20 μm.

(c) Quantification of leading edge length 7 days PWI. N = 3 mice, mean values ± s.e.m.; Tukey corrected one-way ANOVA.

(d) Ki67 immunostaining in control or inhibitor treated mouse skins at 3 and 7 days PWI. Representative confocal images of n = 15 mice, Scale bar: 20 μm.

(e) Percentage of Ki67$^+$ proliferating cells in control and inhibitor treated mouse skins at days 3 and 7 PWI. N = 15 mice, mean values ± s.e.m.; *: $p < 0.0001$ compared to DMSO; Tukey corrected one-way ANOVA.
