# Supplementary material for: Apoptotic stress-induced FGF signalling promotes non-cell autonomous resistance to cell death
Source: Nat Commun. 2021 Nov 12;12:6572. doi: 10.1038/s41467-021-26613-0 (PMC8590049; doi:10.1038/s41467-021-26613-0)
Supplement: Supplementary file 3 — Description of Additional Supplementary Files [file 41467_2021_26613_MOESM3_ESM.pdf]

Description of Additional Supplementary Files

File Name: Supplementary Software 1

Description: Contains the R code to generate the graphs shown in Figure 6f and Supplementary Figures 6a and 6b. The code uses data downloaded from cBioPortal (https://www.cbioportal.org/) of patients with the indicated cancer type to determine the correlation between FGF pathway activation and MCL1 or BCL2 expression as well as patient survival stratified by FGF pathway activation and MCL1 or BCL2 expression.
