# Supplementary material for: Apoptotic stress-induced FGF signalling promotes non-cell autonomous resistance to cell death
Source: Nat Commun. 2021 Nov 12;12:6572. doi: 10.1038/s41467-021-26613-0 (PMC8590049; doi:10.1038/s41467-021-26613-0)
Supplement: Supplementary file 4 — Reporting Summary [file 41467_2021_26613_MOESM4_ESM.pdf]

nature research

Corresponding author(s): Florian J Bock, Stephen WG Tait

Last updated by author(s): Sep 3, 2021

Reporting Summary

Nature Research wishes to improve the reproducibility of the work that we publish. This form provides structure for consistency and transparency in reporting. For further information on Nature Research policies, see our Editorial Policies and the Editorial Policy Checklist.

Statistics

For all statistical analyses, confirm that the following items are present in the figure legend, table legend, main text, or Methods section.

n/a	Confirmed	
☐	☒	The exact sample size (*n*) for each experimental group/condition, given as a discrete number and unit of measurement
☐	☒	A statement on whether measurements were taken from distinct samples or whether the same sample was measured repeatedly
☐	☒	The statistical test(s) used AND whether they are one- or two-sided<br>*Only common tests should be described solely by name; describe more complex techniques in the Methods section.*
☐	☒	A description of all covariates tested
☐	☒	A description of any assumptions or corrections, such as tests of normality and adjustment for multiple comparisons
☐	☒	A full description of the statistical parameters including central tendency (e.g. means) or other basic estimates (e.g. regression coefficient) AND variation (e.g. standard deviation) or associated estimates of uncertainty (e.g. confidence intervals)
☐	☒	For null hypothesis testing, the test statistic (e.g. F, t, r) with confidence intervals, effect sizes, degrees of freedom and P value noted<br>*Give P values as exact values whenever suitable.*
☒	☐	For Bayesian analysis, information on the choice of priors and Markov chain Monte Carlo settings
☒	☐	For hierarchical and complex designs, identification of the appropriate level for tests and full reporting of outcomes
☐	☒	Estimates of effect sizes (e.g. Cohen's d, Pearson's r), indicating how they were calculated

Our web collection on statistics for biologists contains articles on many of the points above.

Software and code

Policy information about availability of computer code

Data collection	- ImageStudio Lite 5.2 (Western Blot)<br>- Quant Studio 3 (qPCR)<br>- Incucyte Zoom/FLR software (Live cell imaging)<br>- BD Cellquest (FACS)
Data analysis	- Flowing 2.5.1 (FACS)<br>- Graph Pad Prism 9 (Graphs and Statistics)<br>- R 4.1.0 (TCGA data analysis)<br>- RStudio 1.4.1106 (TCGA data analysis)

For manuscripts utilizing custom algorithms or software that are central to the research but not yet described in published literature, software must be made available to editors and reviewers. We strongly encourage code deposition in a community repository (e.g. GitHub). See the Nature Research guidelines for submitting code & software for further information.

Data

Policy information about availability of data

All manuscripts must include a data availability statement. This statement should provide the following information, where applicable:

- Accession codes, unique identifiers, or web links for publicly available datasets
- A list of figures that have associated raw data
- A description of any restrictions on data availability

Source data are provided with this paper. The TCGA data used in this study are available in the cBioPortal database under [www.cbioportal.org/study/summary?id=thym_tcga], [https://www.cbioportal.org/study/summary?id=kich_tcga] and [https://www.cbioportal.org/study/summary?id=ucec_tcga]. The FGF score was

based on the fgf2induced dataset from the Harmonizome [https://maayanlab.cloud/Harmonizome/gene_set/fgf2induced/GeneRIF+Biological+Term+Annotations].

Field-specific reporting

Please select the one below that is the best fit for your research. If you are not sure, read the appropriate sections before making your selection.

☒ Life sciences ☐ Behavioural & social sciences ☐ Ecological, evolutionary & environmental sciences

For a reference copy of the document with all sections, see nature.com/documents/nr-reporting-summary-flat.pdf

Life sciences study design

All studies must disclose on these points even when the disclosure is negative.

Sample size	Sample sizes were based on previous experiments, therefore no statistical method was used to predetermine sample size.
Data exclusions	No data was excluded from analysis.
Replication	The number of biologically independent replications are stated in the figure legend.
Randomization	For in vitro experiments, control and treatment groups were derived from the same cell line, so no randomization could be performed.<br><br>For in vivo experiments, mice were randomly assigned to the described treatment groups.
Blinding	No blinding was employed, as the results did not require subjective interpretation.

Reporting for specific materials, systems and methods

We require information from authors about some types of materials, experimental systems and methods used in many studies. Here, indicate whether each material, system or method listed is relevant to your study. If you are not sure if a list item applies to your research, read the appropriate section before selecting a response.

Materials & experimental systems

n/a	Involved in the study	
☐	☒	Antibodies
☐	☒	Eukaryotic cell lines
☒	☐	Palaeontology and archaeology
☐	☒	Animals and other organisms
☒	☐	Human research participants
☒	☐	Clinical data
☒	☐	Dual use research of concern

Methods

n/a	Involved in the study	
☒	☐	ChIP-seq
☐	☒	Flow cytometry
☒	☐	MRI-based neuroimaging

Antibodies

Antibodies used

The following primary antibodies were used: Li-Cor IRDye 800CW donkey anti-rabbit (926-32213, LI-COR Biosciences), Actin (A4700, Sigma), BAK (12105, Cell Signaling), BAX (2772, Cell Signaling), BCL-2 (2762, Cell Signaling), ERK1/2 (4695, Cell Signaling), basic FGF (20102, Cell Signaling), FGFR1 (9740, Cell Signaling), FGFR3 (4574, Cell Signaling), FGFR4 (8562, Cell Signaling), GFP (In house), HSP60 (4870, Cell Signaling), MCL1 (5453, Cell Signaling), pERK1/2 (4370, Cell Signaling), CASPASE 3 (9662, Cell Signaling), CASPASE 9 (9502, Cell Signaling), PARP1 (9532, Cell Signaling) alpha-Tubulin (T5168, Sigma) and cleaved CASPASE 3 (9664, Cell Signaling).

Validation

Actin: Manufacturer validated, 932 citations on www.citeab.com
active ItgB1 : Manufacturer validated, 50 citations on www.citeab.com 50 citations
alpha-Tubulin: Manufacturer validated, 3183 citations on www.citeab.com
BAK: CRISPR Figure S1e
basic FGF: CRISPR Figure S4a
BAX: CRISPR Figure S1e
BCL-2: Validated by siRNA (not shown)
CASPASE 3: Processing during apoptosis Figure S1e
CASPASE 9: Processing during apoptosis Figure S1e
cleaved CASPASE 3: Appearance during apoptosis Figure S1e
ERK1/2: CRISPR Figure 3f
FGFR1: siRNA Figure 4g
FGFR3: siRNA Figure S4f

FGFR4: siRNA Figure 4g
GFP: Overexpression Figure S1h
HSP60: Manufacturer validated, 75 citations on www.citeab.com
Ki67 : Manufacturer validated, 42 citations on www.citeab.com
MCL1: Validated by CRISPR (not shown)
Mcm2 : Manufacturer validated, 153 citations on www.citeab.com
PARP1: Processing during apopotosis Figure S1e
pERK1/2: Upstream kinase inhibitor Figure 3b

Eukaryotic cell lines

Policy information about cell lines

Cell line source(s)	HeLa: ATCC<br>HeLa tBID2A BCL-2: Ref 9<br>IMR90: Gift from Peter Adams, Beatson Institute<br>MRC5: Gift from Peter Adams, Beatson Institute<br>293T: ATCC<br>CWR-R1: Gift from Arnaud Blomme, Beatson Institute
Authentication	Cells lines were not authenticated
Mycoplasma contamination	Cell lines routinely tested negative for mycoplasma contamination.
Commonly misidentified lines (See ICLAC register)	No Commonly misidentified lines were used.

Animals and other organisms

Policy information about studies involving animals; ARRIVE guidelines recommended for reporting animal research

Laboratory animals	8-12-weeks-old sex and age matched male and female C57BL/6J mice were used. Mice were housed in specific pathogen free (SPF) conditions, dark/light cycles: 13-hour light/11-hour dark, ambient temperature 23 C and humidity 53%.
Wild animals	Study did not involved wild animals.
Field-collected samples	Study did not involve samples collected in the field.
Ethics oversight	Housing, care and wounding experiments were approved by the ethical committee of the Technion - Israel Institute of Technology.

Note that full information on the approval of the study protocol must also be provided in the manuscript.

Flow Cytometry

Plots

Confirm that:

- [x] The axis labels state the marker and fluorochrome used (e.g. CD4-FITC).
- [x] The axis scales are clearly visible. Include numbers along axes only for bottom left plot of group (a 'group' is an analysis of identical markers).
- [x] All plots are contour plots with outliers or pseudocolor plots.
- [x] A numerical value for number of cells or percentage (with statistics) is provided.

Methodology

Sample preparation	Adherent cell lines were harvested by trypsinization, washed in PBS and stained for AnnexinV and propidium iodide
Instrument	FACSCalibur (BD Biosciences, San Jose, CA)
Software	Flowing Software version 2.5.1
Cell population abundance	Alive cells (AnnexinV and propidium iodide negative) were part of a pure population of HeLa tBID2A BCL-2 cells
Gating strategy	Whole cells were gated using FCS and SSC, and alive cells were classified as Annexin V-/ propidium iodide-. The gating strategy is also provided in the Supplementary Information.

- [x] Tick this box to confirm that a figure exemplifying the gating strategy is provided in the Supplementary Information.
