# Supplementary material for: Apoptotic stress-induced FGF signalling promotes non-cell autonomous resistance to cell death
Source: Nat Commun. 2021 Nov 12;12:6572. doi: 10.1038/s41467-021-26613-0 (PMC8590049; doi:10.1038/s41467-021-26613-0)

Source Files

1c

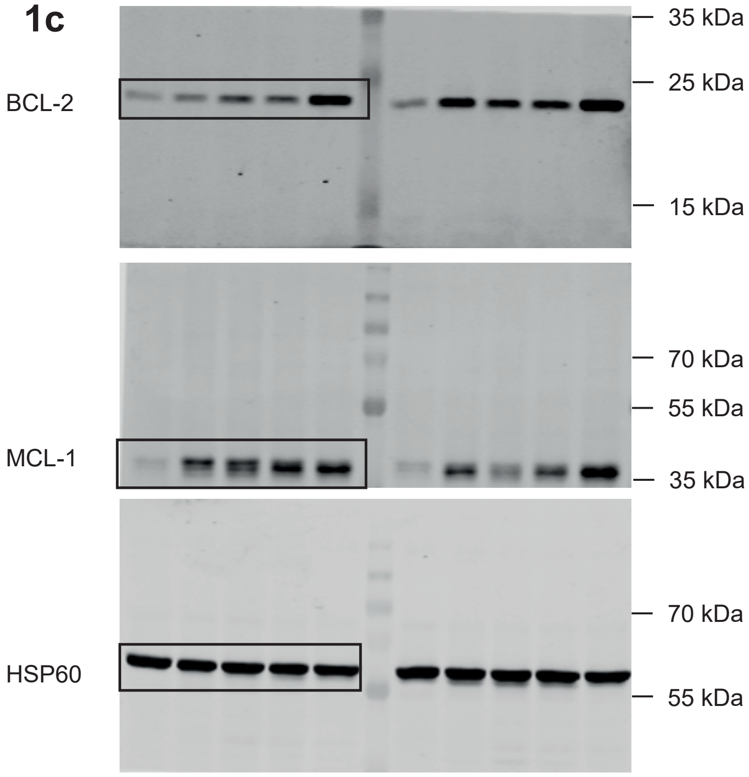

1d

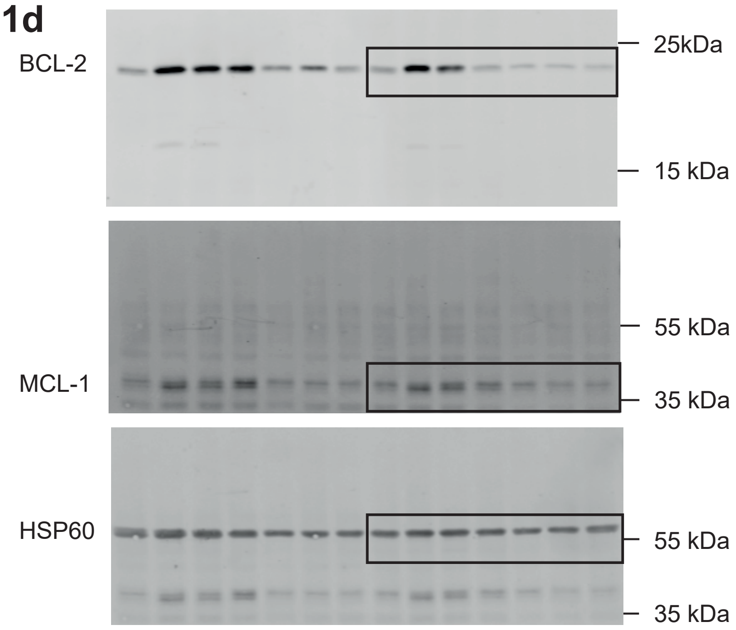

1f

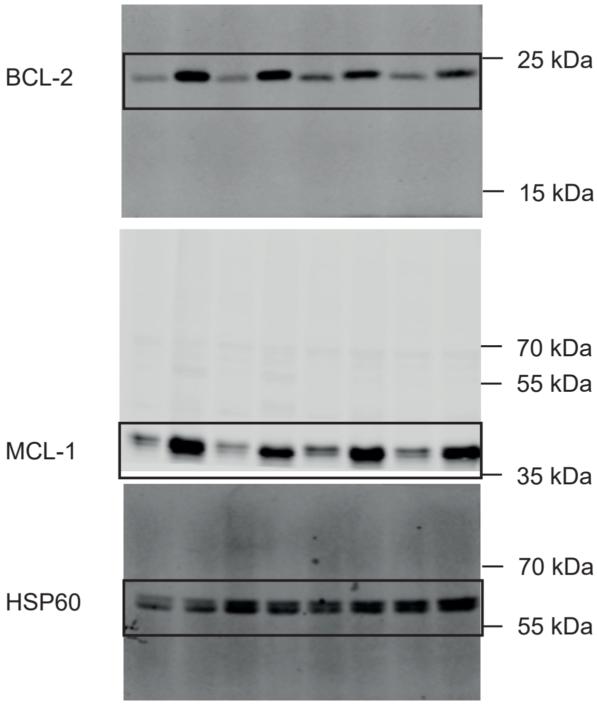

1g

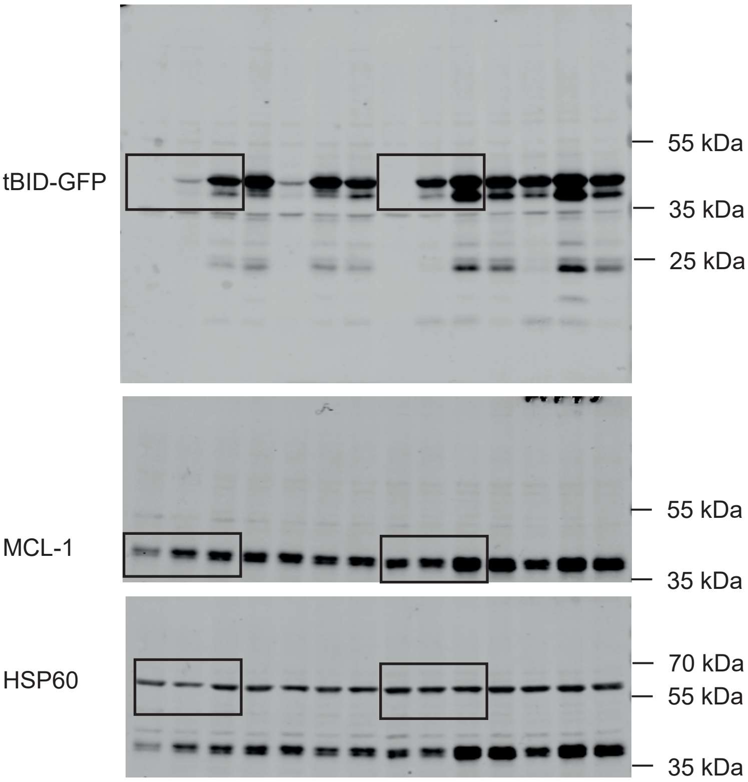

Source Files

2b

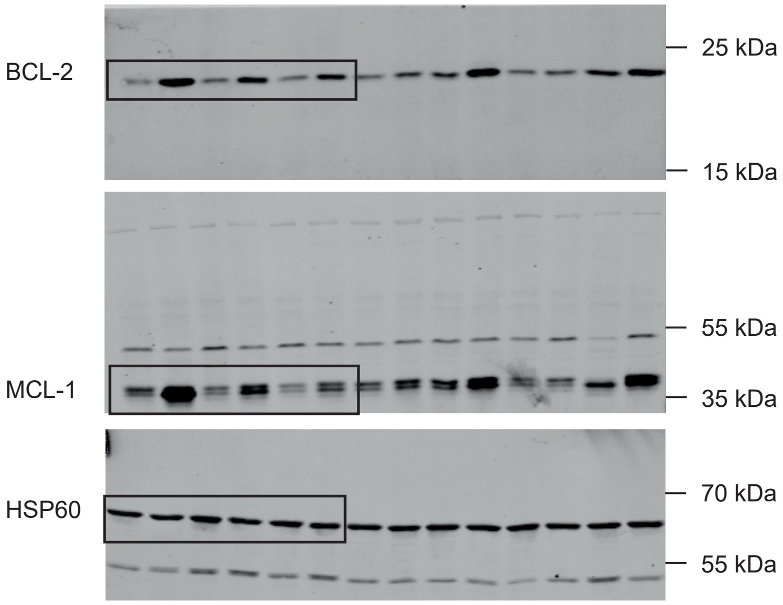

2c

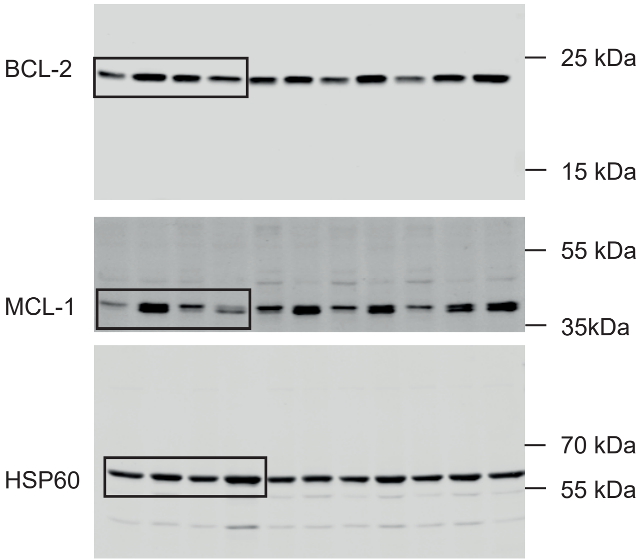

2d

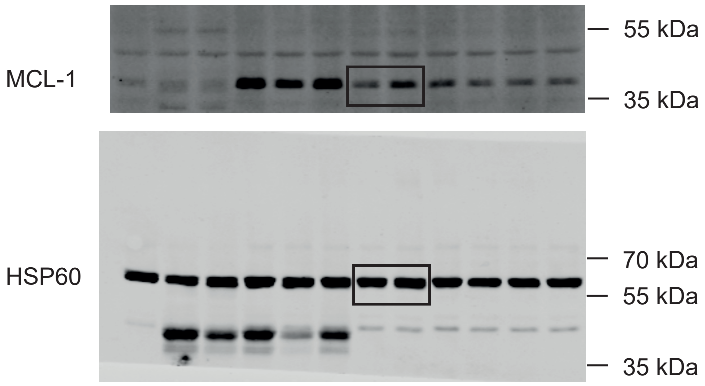

Source Files

3a

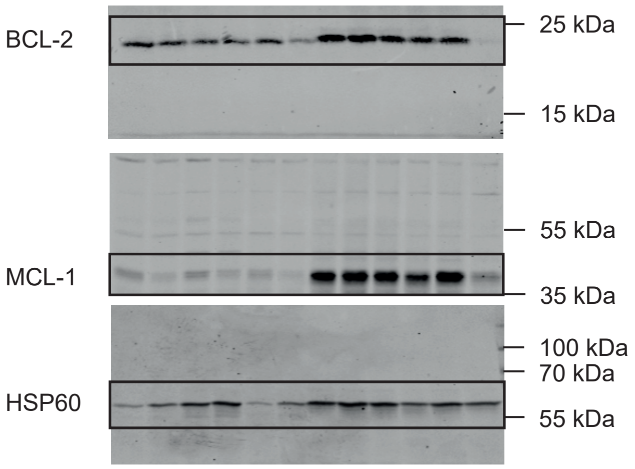

3b

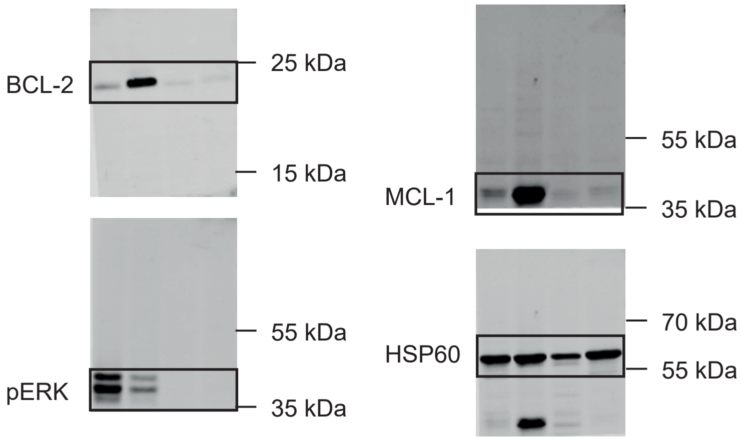

3d

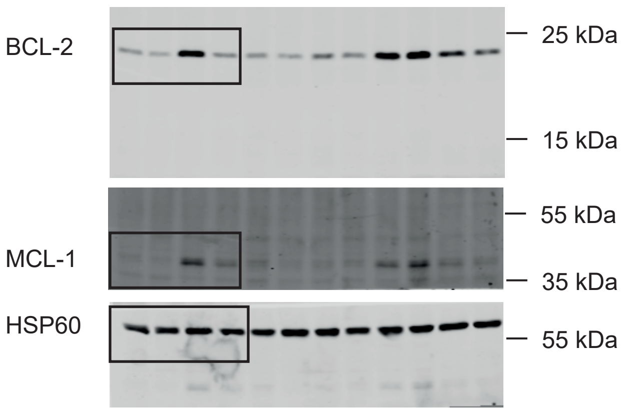

3e

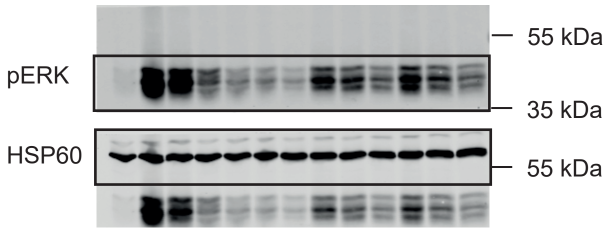

3h

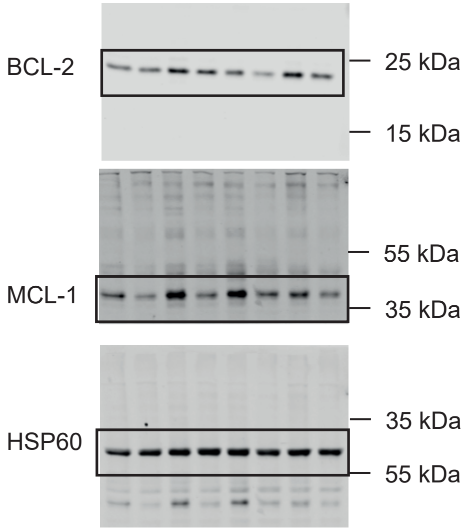

3f

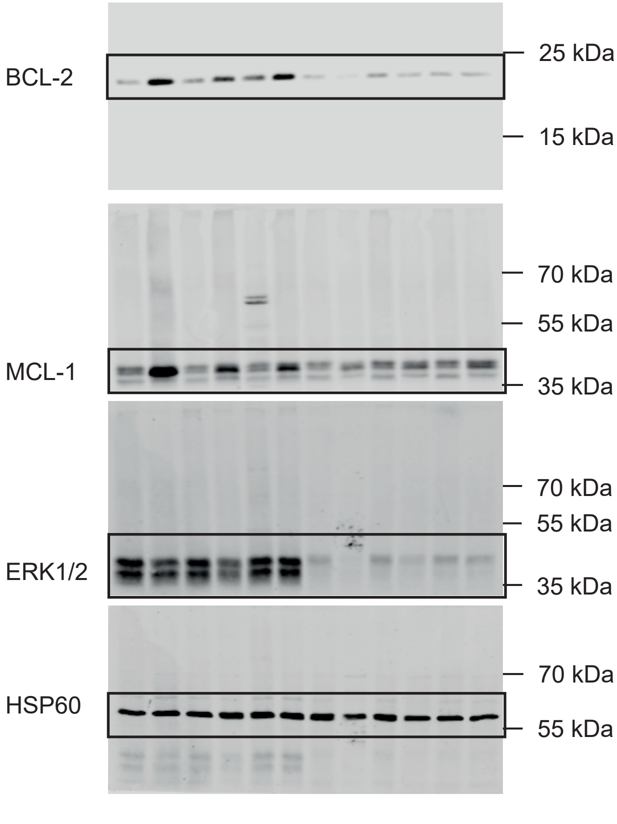

Source Files

4c

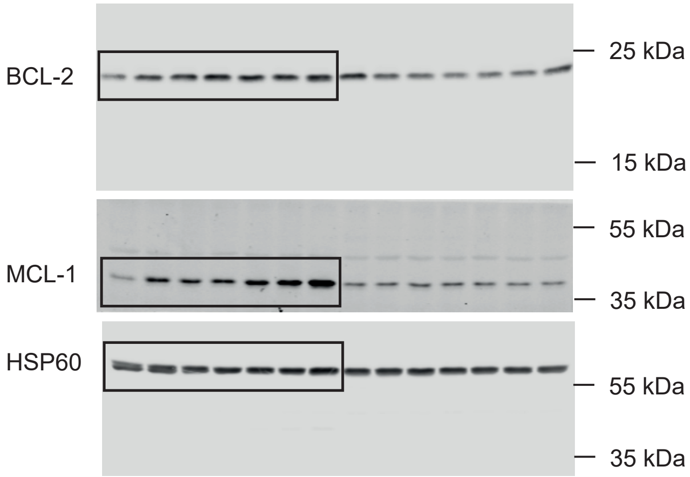

4d

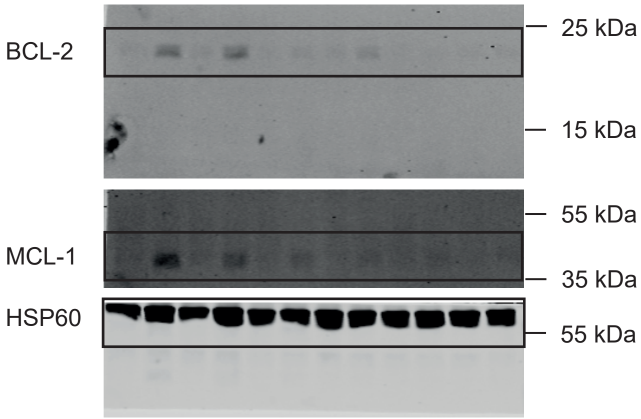

4f

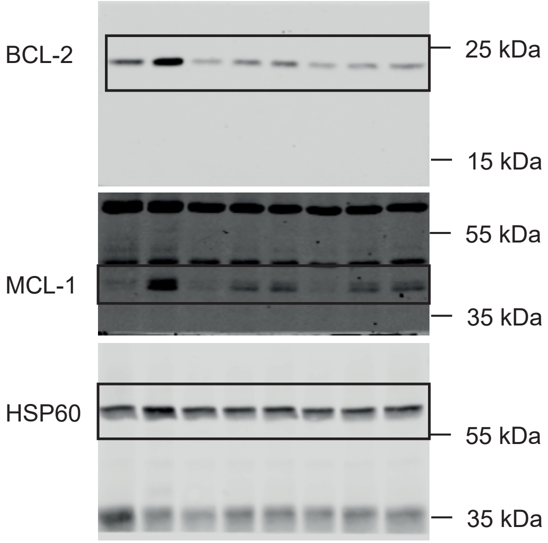

4g

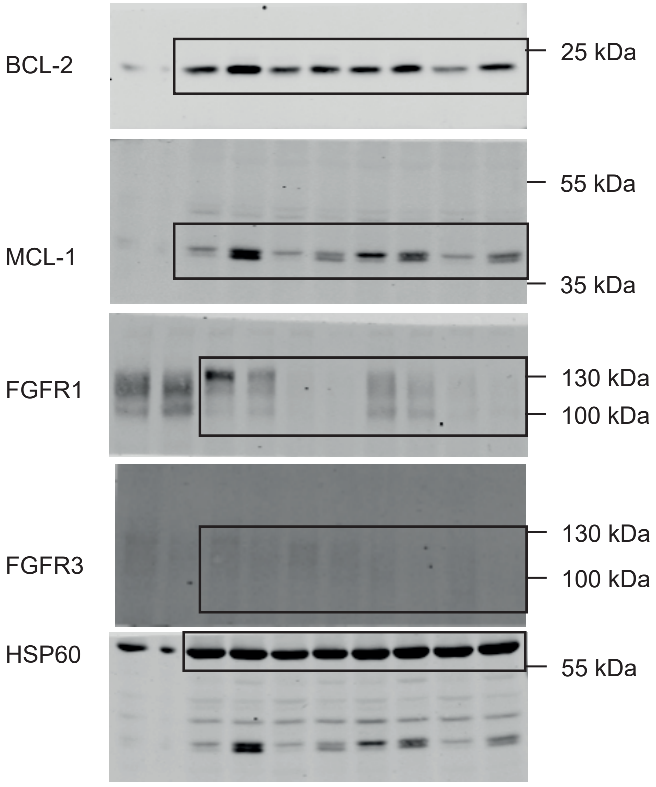

4h

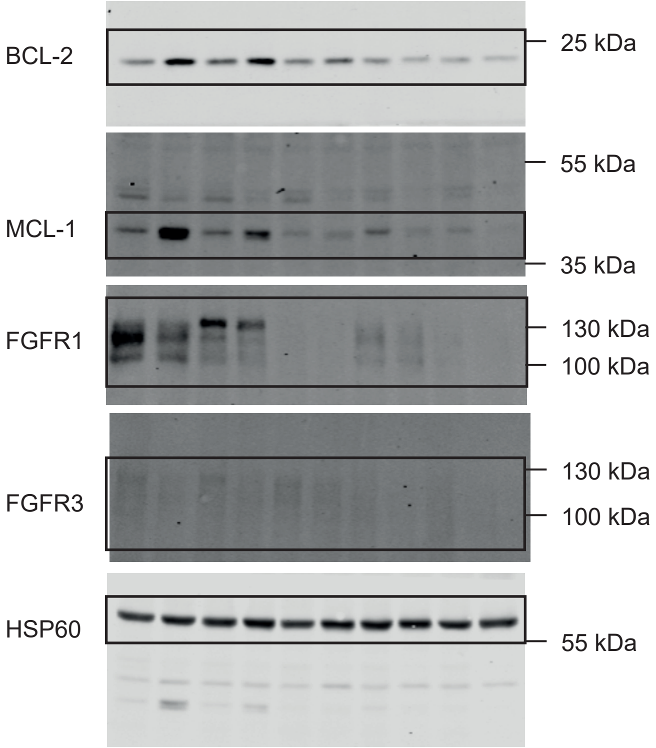

Source Files

5b

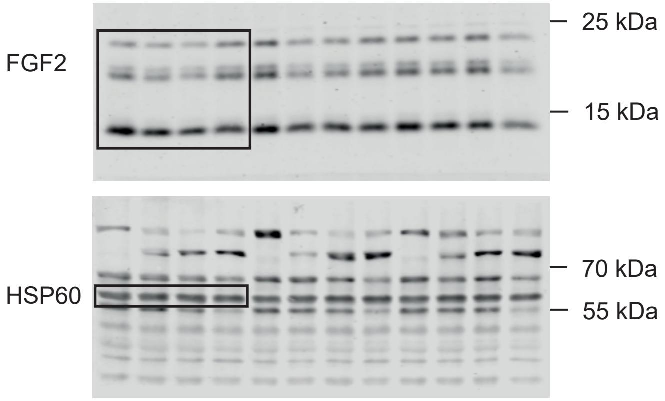

5c

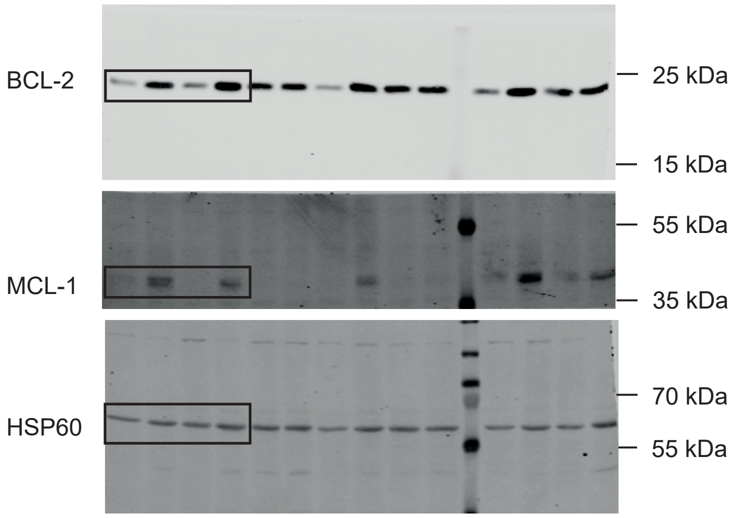

5d

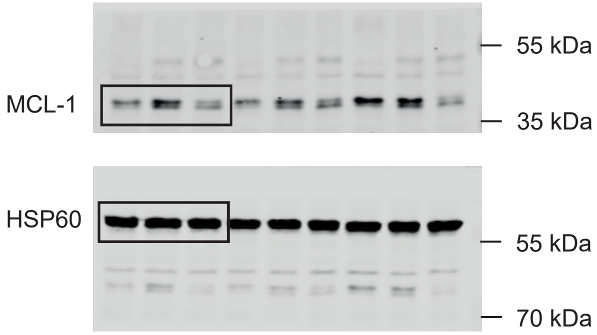

Source Files

S1a

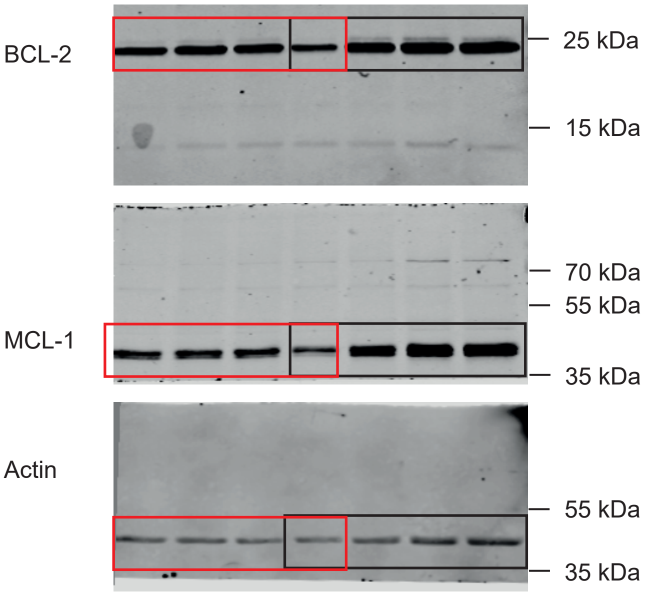

Red: Blot on the right in Supplementary Figure 1a
Black: Blot on the left in Supplementary Figure 1a

S1c

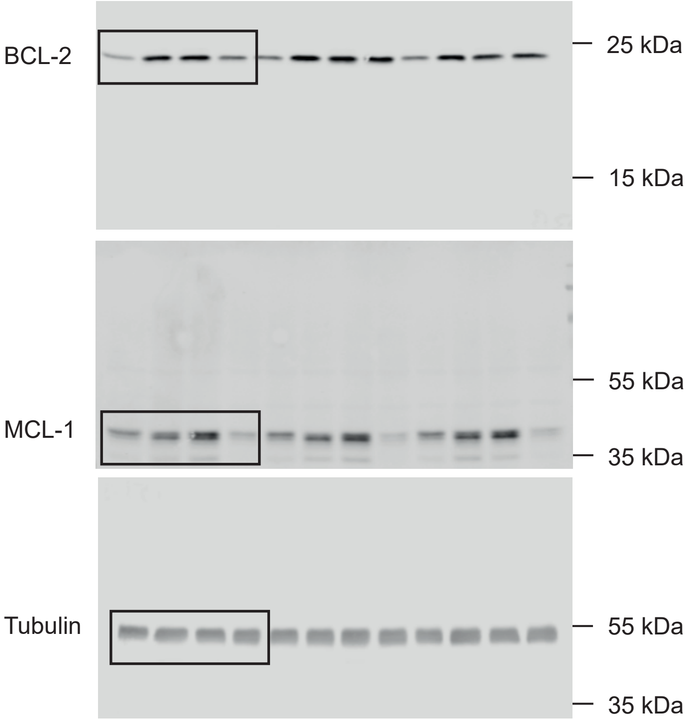

S1d

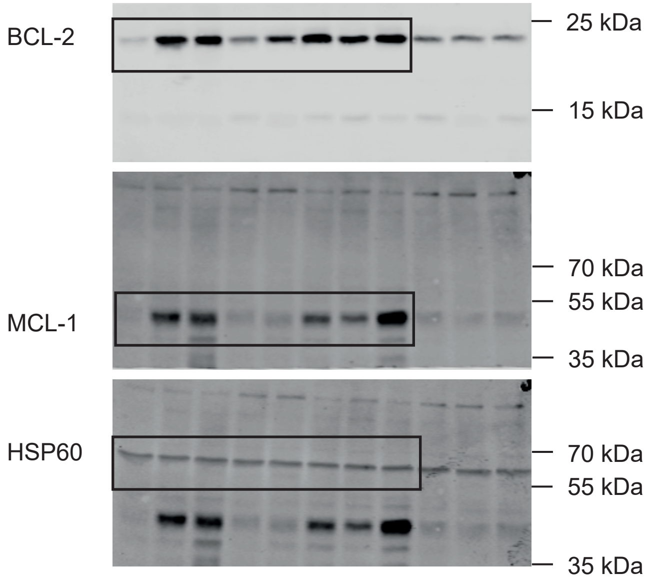

Source Files

S1e

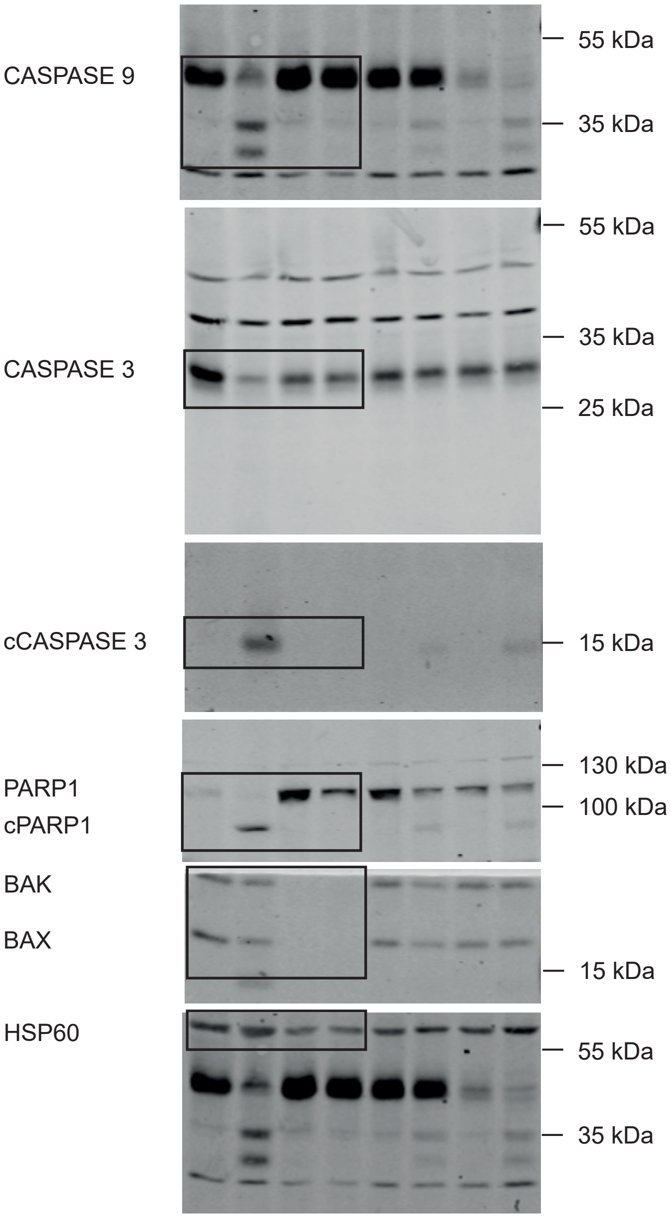

S1g

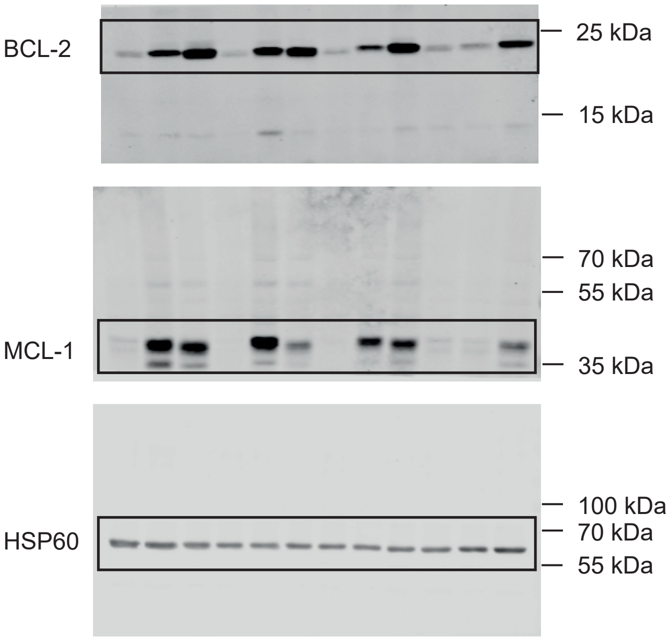

S1h

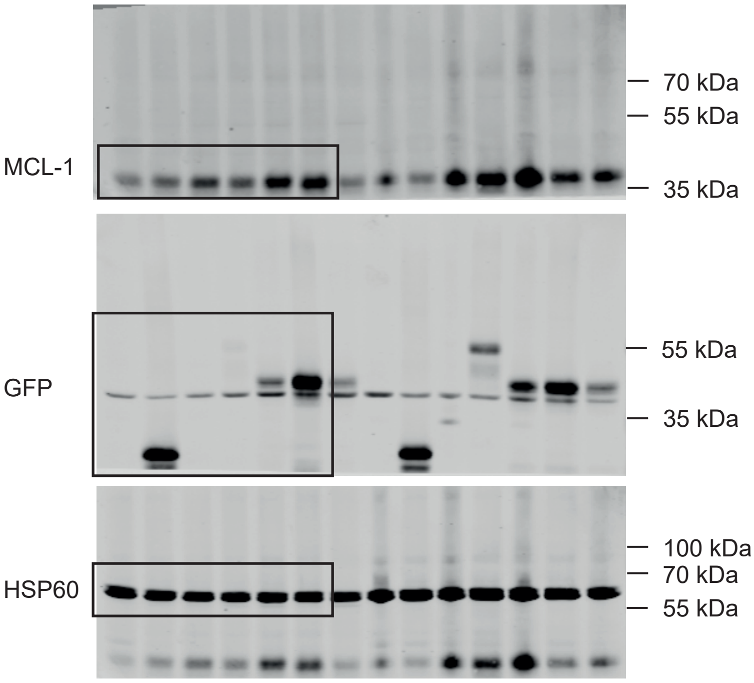

Source Files

S3a

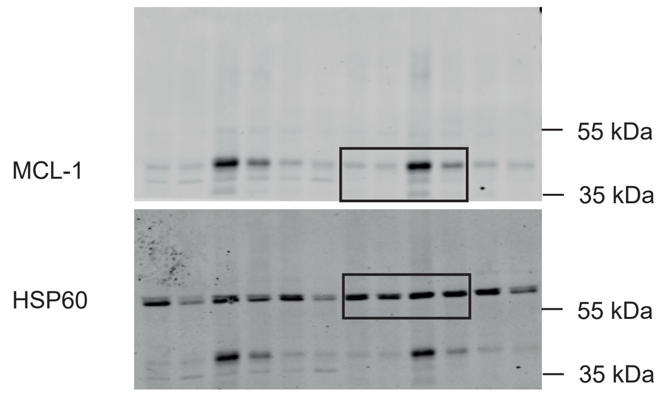

S3b

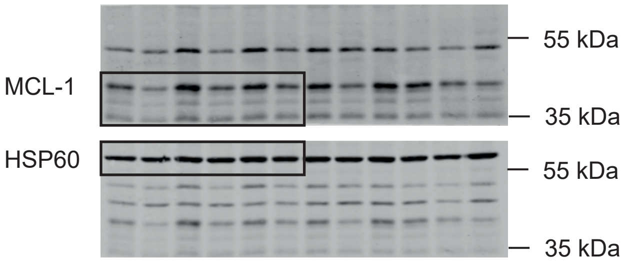

S3c

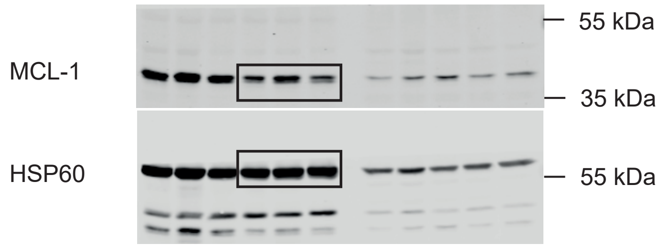

Source Files

S4a

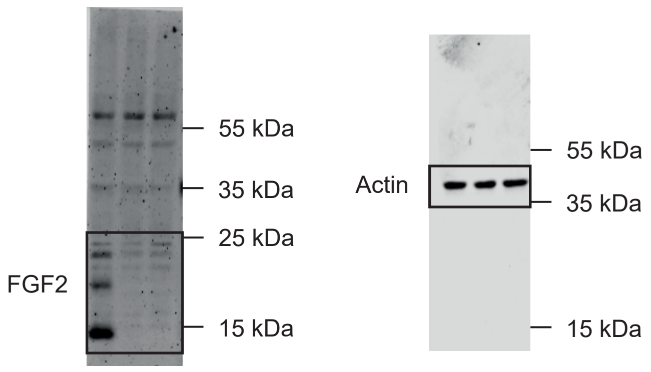

S4b

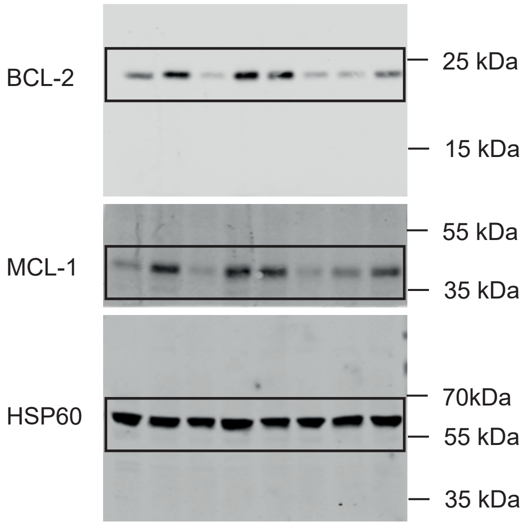

S4c

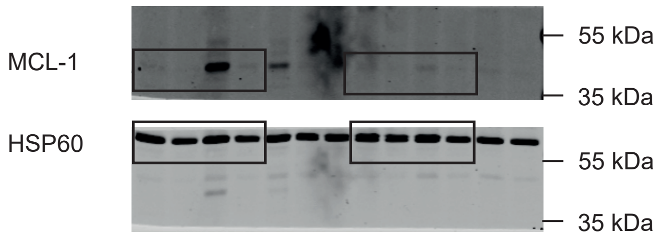

S4f

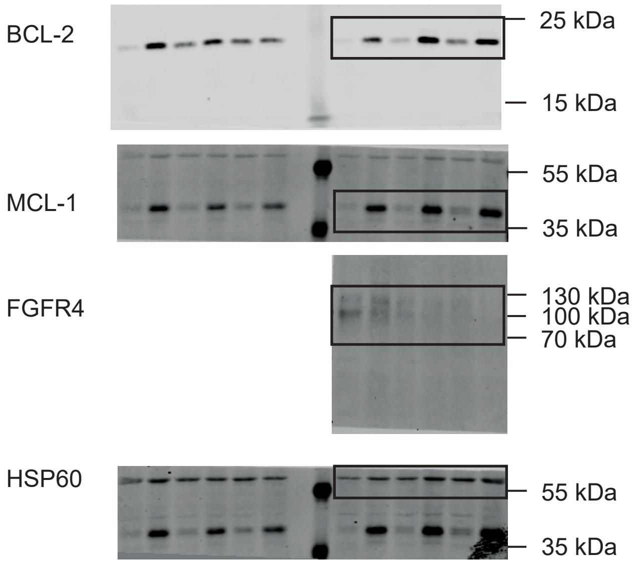

S4g

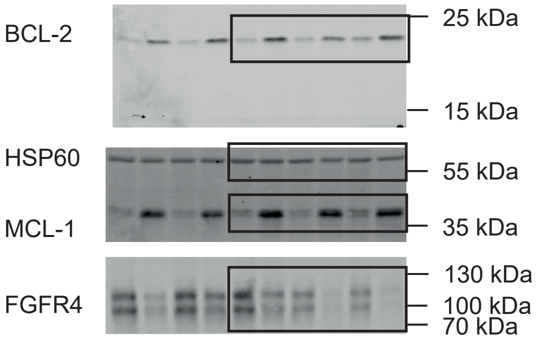

Supplement: Supplementary file 5 — Source Data [file 41467_2021_26613_MOESM5_ESM.pdf]
